# Water-Soluble Coffee Melanoidins Inhibit Digestive Proteases

**DOI:** 10.1021/acs.jafc.3c09654

**Published:** 2024-03-08

**Authors:** Jana Raupbach, Antonio Dario Troise, Vincenzo Fogliano

**Affiliations:** † Department of Molecular Toxicology, German Institute of Human Nutrition Potsdam-Rehbruecke (DIfE), 14558 Nuthetal, Germany; ‡ Proteomics, Metabolomics & Mass Spectrometry Laboratory, Institute for the Animal Production System in the Mediterranean Environment, National Research Council, 80055 Portici, Italy; § Food Quality & Design Group, Wageningen University & Research, Wageningen, NL-6708 WG, Netherlands

**Keywords:** coffee, melanoidins, pepsin, trypsin, digestion

## Abstract

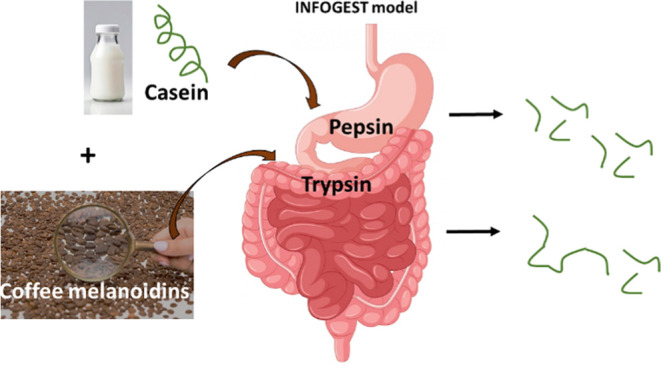

Coffee is one of the most popular beverages around the world and its consumption contributes to the daily intake of dietary melanoidins. Despite the emerging physiological role of food melanoidins, their effect on digestive processes has not been studied so far. In this study, the activity of the gastrointestinal enzymes pepsin and trypsin was investigated in the presence of water-soluble coffee melanoidins. The gastric enzyme pepsin is only slightly affected, whereas the intestinal enzyme trypsin is severely inhibited by coffee melanoidins. The intestinal digestibility of casein was significantly inhibited by coffee melanoidins at a concentration achievable by regular coffee consumption. The inhibition of proteolytic enzymes by coffee melanoidins might decrease the nutritional value of dietary proteins.

## Introduction

Human gastrointestinal digestion is relevant to the proper utilization of dietary proteins. The digestive process includes oral, gastric, and intestinal phases and large intestinal fermentation. Protein digestion mainly occurs during gastric and intestinal phases, whereby amino acids are taken from the protein and released into the bloodstream.^1^ The digestibility of proteins depends on factors that may be internal and external to the protein. Internal factors include protein amino acid profile, protein folding, and cross-linking. External factors include pH, temperature, ionic strength conditions, and efficacy of proteases. The presence of other food components influences the texture of the food matrix and can increase or decrease the digestibility.^2,3^ For the digestion of protein of dry fractionated quinoa seeds, it was shown that the presence of fiber and starch from whole quinoa flour significantly decreased gastric protein digestibility.^4^ Another study demonstrated that the presence of dietary fat substantially improved the digestibility of pork protein (from 80 to 86%) and chicken protein (from 69 to 87%) but had no effect on casein and soy protein.^5^ These studies emphasize that human gastrointestinal digestion is a complex, dynamic process and that the composition of our diet markedly influences the nutritional value of its components.

Food processing and its potential reaction products might have a considerable effect on protein digestibility. One reaction that is significantly influenced during the thermal treatment of food is the Maillard reaction, which describes the nonenzymatic reaction between reducing sugars and amino compounds. The Maillard reaction does not refer to one specific reaction but involves a reaction cascade leading to a plethora of structurally diverse compounds.^6,7^ Melanoidins, which are complex nitrogen-containing browning products, are massively formed during the late stage of the reaction. They are characterized by a heterogeneous chemical nature and are often found as brown pigments in heated foods.^8^ Their actual molecular weight ranges from a few thousand Da (premelanoidins^9^) to 10–100 kDa.^10^ Melanoidins are rather hydrophilic and negatively charged. Prominent examples for melanoidin-containing food items are bakery products, coffee, and beer. With a diet rich in bread, biscuits, coffee and cereals, up to 10 g of melanoidins are ingested daily.^11^ The structure of melanoidins and therefore their physiological effects are highly dependent on the reactants. In some foods, melanoidins are protein-based (melanoproteins), while in coffee, melanoidins are rich in polysaccharides and polyphenols.^8^ Due to their high-molecular-weight and their resistance to proteolytic cleavage, it is unlikely that dietary melanoidins are absorbed during gastrointestinal digestion.^12,13^ Mainly beneficial physiological effects, such as antioxidative,^14^ anticariogenic,^15^ antimicrobial,^16^ and antiglycative^17^ effects of high-molecular-weight coffee melanoidins are described so far. Coffee melanoidins induce Nrf2-mediated antioxidative responses by alteration of detoxifying enzyme activities in the liver of experimental rats,^18^ while Vitaglione and co-workers showed a positive effect of coffee melanoidins on liver steatosis.^19^ Moreover, it was shown that coffee constituents are modulators of phase I and II enzymes which play a pivotal role in the biotransformation of xenobiotics.^20^ Besides the many potentially positive health effects shown for coffee melanoidins, Muscat et al.^21^ outlined that coffee induces NFκB translocation in macrophages via the generation of hydrogen peroxide. Since the authors worked with whole coffee preparations, it is unclear whether melanoidins or other coffee fractions such as low-molecular-weight compounds and polyphenols are responsible for this effect.

Few papers investigated the direct effect of coffee melanoidins on the digestion of other food components, particularly proteins. Ibarz et al.^22^ reported that melanoidins extracted from a heat-treated glucose–asparagine mixture inhibited the gastrointestinal enzyme trypsin by approximately 30% at a concentration of 150 μg/mL. Due to their high-molecular- weight and their limited absorption, it is assumed that the gastrointestinal tract is the major site of biological action of dietary melanoidins^13^ and their concentration can be relatively high upon consumption of coffee together or right after the main meals. The aim of this study was to analyze the effect of dietary melanoidins derived from coffee on individual gastric and intestinal proteolytic enzymes. Additionally, the effect of coffee melanoidins on protein digestibility by using a static in vitro simulation of gastrointestinal digestion was studied.

## Materials and Methods

### Chemicals

Chemicals and enzymes used were standard analytical grade and were purchased from Sigma-Aldrich Inc. (St. Louis, MO) unless stated otherwise. Pepsin (P7012), bile (B8631), and pancreatin (P7545; 8 × USP specifications activity) were all of porcine origin. Sodium caseinate was purchased from Meggle (Wasserburg, Germany). Dichloromethane was obtained from Merck (Darmstadt, Germany). Liquid chromatography solvents were of mass spectrometry grade and were obtained from Merck (Darmstadt, Germany).

### Extraction of Water-Soluble Coffee Melanoidins

Preparation of high-molecular-weight coffee melanoidins was performed as previously described.^17^ The brewing conditions used for the extraction of soluble melanoidins was chosen to obtain high amounts of soluble melanoidins and do not resemble a domestic coffee preparation. Ground medium-light-roasted coffee powder containing 100% arabica coffee beans from regions such as South America, Latin America, and India was purchased from Roast Market GmbH, Germany. A dose of 245 g of coffee powder was defatted by washing three times with 400 mL of dichloromethane and was subsequently dried for 3 h at room temperature. The defatted, dried coffee powder was brewed with 1 L of water at 80 °C. After 20 min of incubation, the coffee brew was filtered (Whatman prepleated qualitative filter paper, grade 595), and the eluate was dialyzed against water for 3 days (MWCO 14 kDa). Dialysis water was changed two times a day. After dialysis, the concentrated coffee brew containing water-soluble, high-molecular-weight compounds (>14 kDa) was lyophilized. The lyophilized high-molecular-weight coffee melanoidins were stored at 4 °C until further use.

### Simulated Digestion Model

In vitro simulated digestion was performed according to the INFOGEST protocol.^23,24^ The enzyme activities were measured before the digestion experiment as described previously.^23^ In brief, an aliquot of 5 mL of sample was mixed with 5 mL of simulated salivary fluid (pH 7, 37 °C) and incubated at 37 °C for 2 min. Then, 10 mL of simulated gastric juice (pH 3, 37 °C) containing pepsin (2000 U/mL of digesta) was added and incubated for 120 min. Subsequently, 20 mL of simulated intestinal juice (pH 7, 37 °C) containing pancreatin (100 U trypsin activity/mL of digesta) and bile (10 mM of total digesta) was added and incubated for 120 min. The digestion protocol was performed at 37 °C under constant gentle mixing in an overhead shaker. Simulated digestion was stopped by heating the samples at 100 °C for 5 min. Samples were taken directly after mixing the sample with simulated salivary fluid (*t*_0_), after the gastric phase (GP), and after the intestinal phase (IP). Immediately after stopping the digestion, all samples were frozen at −20 °C. For digestion, casein from cow’s milk (20 mg/mL) was used individually and in the presence of water-soluble coffee melanoidins (1 mg/mL). A blank sample containing water or water-soluble coffee melanoidins (1 mg/mL) was included in the analysis.

### Analysis of Free Amino Compounds

The free amino groups were measured using the o-phthaldialdehyde (OPA) assay according to Opazo-Navarrete et al.^25^ with slight modifications. Briefly, an aliquot of 200 μL digestion sample (*t*_0_, GP, and IP) was mixed with 1.5 mL of OPA reagent. The mixture was incubated for 2 min at room temperature before the absorbance was read at 340 nm. The OPA reagent (100 mL) was prepared by dissolving 3.81 g of sodium tetraborate decahydrate (Borax) and 0.1 g of SDS in 80 mL of milli-Q water. Along with 88 mg of dithiothreitol (DTT), 2 mL of an OPA (40 mg/mL in ethanol) solution was added to the Borax–SDS solution. The solution was filled up to 100 mL with milli-Q water and used within 2 h. Digestion samples were prediluted in purified water before analysis as follows: *t*_0_ was diluted 1:10, GP was diluted 1:50, and IP was diluted 1:100. A standard curve was prepared using l-serine in a concentration range of 8–170 mg/L. Free amino groups were expressed as serine amino equivalents (mM).

### Analysis of Amino Acids

Amino acids were analyzed by liquid chromatography with high-resolution tandem mass spectrometry through a Vanquish Core system coupled to a quadrupole Orbitrap (Exploris 120, Thermo Fisher Scientific, Bremen, Germany). Aqueous supernatants were diluted in acetonitrile/water 50:50 v/v, and 1 μL was directly injected in full loop mode. Amino acids were separated at 35 °C through a sulfobetaine zwitterionic column (Syncronis HILIC, 100 μm × 2.1, 1.7 μm, Thermo Fisher Scientific). Mobile phases consisted of 0.1% formic acid in acetonitrile (A) and 0.1% formic acid in water (B), running at a rate of 0.3 mL/min. The following gradient of solvent B was used (minutes/%B): (0/5), (2/5), (6/60), (9/95), and (13/95). For positive ion mode, H-ESI interface parameters were as follows: spray voltage 3.3 kV; ion transfer tube and vaporizer gas temperatures were both at 280 °C; sheath gas flow and auxiliary gas flow were set at 40 and 10 (arbitrary units), respectively. Amino acids were quantified in product ion scan mode screening the precursor ions according to a mass list generated in the Trace Finder environment (v. 5.1, Thermo Fisher, Table 1). A set of calibration curves of pure reference amino acid standards was prepared in the range of 0.1–10 μM (Merck-Sigma-Aldrich, Darmstadt, Germany). For amino acid confirmation in product ion scan mode, normalized collision energy was set at 30%, Orbitrap resolution was set at 30,000 (fwhm at *m*/*z* 200), and the quadrupole resolution was set at 1. Profile data were collected using Xcalibur 4.5 (Thermo Fisher Scientific), and analytical performances were monitored according to Troise et al.^26^

**Table 1:** Amino Acids were Quantified in Positive Ion Mode in Full Scan and Product Ion Scan Upon HILIC Separation^a^

compound	formula	precursor (*m*/*z*)	Δ*p*pm
**Glycine**	C2H5NO2	76.0393	–1.0
**Alanine**	C3H7NO2	90.0550	–1.2
**Serine**	C3H7NO3	106.0499	–1.1
**Proline**	C5H9NO2	116.0706	–1.0
**Valine**	C5H11NO2	118.0863	0.8
**Threonine**	C4H9NO3	120.0655	0.8
**Cysteine**	C3H7NO2S	122.0270	0.2
**Isoleucine/Leucine**	C6H13NO2	132.1019	0.1
**Asparagine**	C4H8N2O3	133.0608	–0.5
**Aspartic acid**	C4H7NO4	134.0448	0.4
**Glutamine**	C5H10N2O3	147.0764	0.3
**Lysine**	C6H14N2O2	147.1128	0.1
**Glutamic acid**	C5H9NO4	148.0604	–1.1
**Methionine**	C5H11NO2S	150.0583	1.0
**Histidine**	C6H9N3O2	156.0768	1.0
**Phenylalanine**	C9H11NO2	166.0863	–0.5
**Arginine**	C6H14N4O2	175.1190	–0.9
**Tyrosine**	C9H11NO3	182.0812	1.0
**Tryptophan**	C11H12N2O2	205.0972	–0.2

a Precursor ions [M + H]^+^ were identified with a mass accuracy below 3 ppm (Δ ppm). The amino acids isoleucine and leucine were not separated in sample runs and hence quantified as a single peak.

### Analysis of Pepsin Activity

Enzyme activity was analyzed according to Anson et al.^27^ In brief, an aliquot of 450 μL of the substrate hemoglobin (20 mg/mL in purified water, adjusted to pH 2 with 300 mM HCl) was preincubated in a shaking incubator at 37 °C for 5 min. After reaching the assay temperature, 50 μL of water or melanoidin solution and 100 μL of pepsin solution were added and incubated for exactly 10 min. For pepsin solutions, a stock solution (1 mg/mL in 10 mM Tris buffer and 150 mM NaCl at pH 6.5) was prepared. The stock solution was stored on ice or refrigerated at 4 °C. Just before the assay, the pepsin stock solution was diluted to 20 μg/mL in 10 mM HCl and used for the assay. For the melanoidin solution, a stock solution was prepared in purified water (6 mg/mL). An aliquot (50 μL) of the undiluted stock solution or a dilution ranging from 0.06 to 2.40 mg/mL was added to the assay mixture. The reaction was stopped by adding 1 mL of TCA (5%) to each sample. After centrifugation at 10,000 rpm at 4 °C for 15 min, the absorption of the supernatant was analyzed at 280 nm. A blank sample was acquired by following the same procedure but adding pepsin after the addition of TCA. Enzyme activity (unit per milligram) was calculated using the following formula
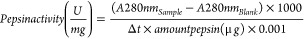
The concentration of a water-soluble coffee melanoidin needed to inhibit 50% of pepsin activity (IC_50_) was determined by plotting the percentage of pepsin inhibition against the logarithm of the different concentrations of water-soluble coffee melanoidins. The fitting of the sigmoid dose–response curve was performed using a nonlinear fit (log(inhibitor) vs response) with Graph-Pad Prism version 9.2.0 (San Diego).

### Analysis of Trypsin Activity

Enzyme activity was analyzed according to Hummel.^28^ In brief, 1300 μL of assay buffer (46 mM Tris/HCl buffer containing 11.5 mM CaCl_2_ at pH 8.1) mixed with 150 μL of substrate Nα-p-tosyl-l-arginine-methyl ester hydrochloride (TAME, 10 mM in purified water) was preincubated at room temperature for 5 min. Subsequently, 50 μL of trypsin solution (15 μg/mL in 1 mM HCl) was added, and absorption at 247 nm was recorded continuously for 10 min. To study the inhibition of trypsin by water-soluble coffee melanoidins, a stock solution of coffee melanoidins (1 mg/mL in assay buffer) was diluted in assay buffer to concentrations ranging from 0.005 to 0.8 mg/mL. For inhibition studies, an assay buffer containing different concentrations of melanoidins was added to the samples. To obtain a blank value, trypsin solution was replaced by 50 μL of 1 mM HCl. To calculate trypsin activity, a slope Δ*A*_247_ was calculated between 2 and 7 min of incubation. Enzyme activity (U/mg) was calculated using the following formula
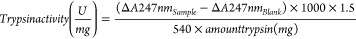
The concentration of a water-soluble coffee melanoidin needed to inhibit 50% of trypsin activity (IC_50_) was determined by plotting the percentage of trypsin inhibition against the logarithm of the different concentrations of water-soluble coffee melanoidins. The fitting of the sigmoid dose–response curve was performed using a nonlinear fit (log(inhibitor) vs response) with Graph-Pad Prism version 9.2.0 (San Diego).

### Statistical Analysis

Statistical analysis was performed by using Graph-Pad Prism version 9.2.0 (San Diego). The Shapiro–Wilk normality test was used to assess normal distribution. Group comparisons were performed by Student’s *t* test analysis. All data are presented as mean values ± SD. Statistically significant differences were considered if *p* < 0.05 and marked with * for *p* values ≤0.05, with ** for *p* values ≤0.01, and with *** for *p* values ≤0.001.

## Results and Discussion

**Figure 1:**
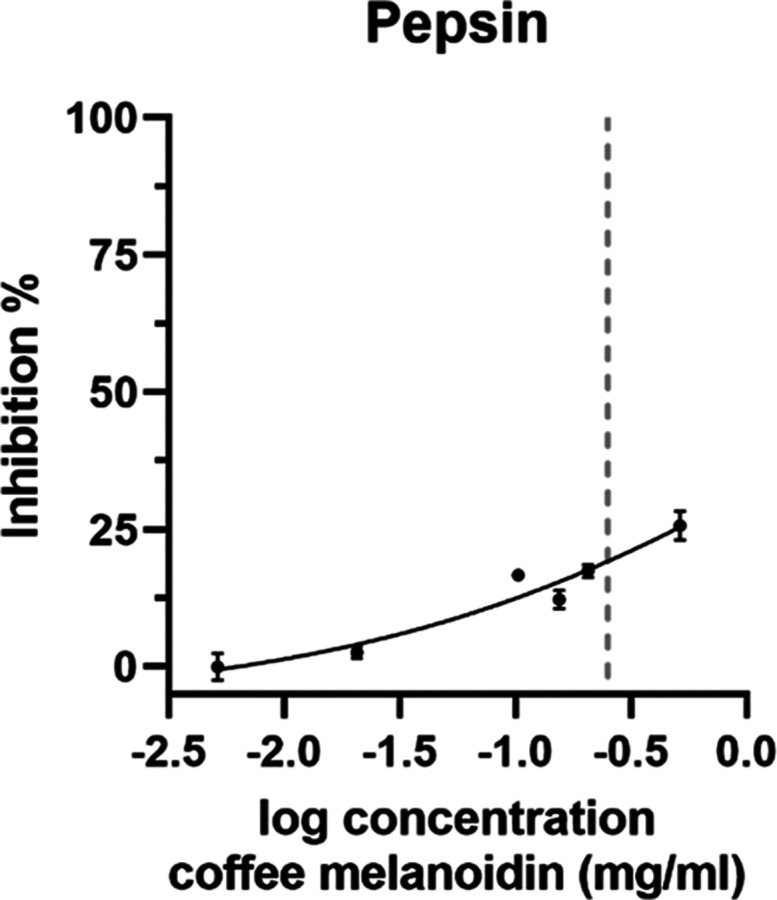
Inhibition of pepsin with water-soluble coffee melanoidins. The dose–response curve of pepsin and water-soluble coffee melanoidins. The dotted line marks the concentration present in the simulated gastric digestion experiment (0.25 mg/mL). Data are mean ± S.D., *n* = 3.

Coffee melanoidins were isolated from coffee brew via dialysis (14 kDa cutoff tubes), and the activity of gastrointestinal enzymes pepsin and trypsin was analyzed in their presence and absence. The dose–response curve of pepsin and coffee melanoidins ranging in concentrations from 0.005 to 0.5 mg/mL is shown in Figure 1. With the highest concentration tested in this assay (0.5 mg/mL), the pepsin activity is inhibited by approximately 25%. To reach this concentration in the assay, we used the melanoidin stock solution with a concentration of 6 mg/mL. Concentrations above 0.5 mg/mL could not be tested, since the extracted melanoidins showed limited solubility with a maximum soluble amount of 6 mg/mL in the stock solution. The average daily intake of coffee melanoidins was estimated between 0.5 and 2 g per day.^11^ Assuming that the complete daily dose reaches the stomach in one portion, this would equal a maximum gastric concentration of 0.3–1.3 mg/mL in 1.5 L of stomach fluid.^29^ According to the nonlinear fit of the dose–response curve of pepsin, the concentration of water-soluble coffee melanoidins needed to inhibit 50% of pepsin activity (IC_50_ value) is approximately 3 mg/mL. Therefore, we hypothesize that the slight inhibition of pepsin in the presence of coffee melanoidins observed in vitro has very limited impact on proteolytic degradation in humans.

**Figure 2:**
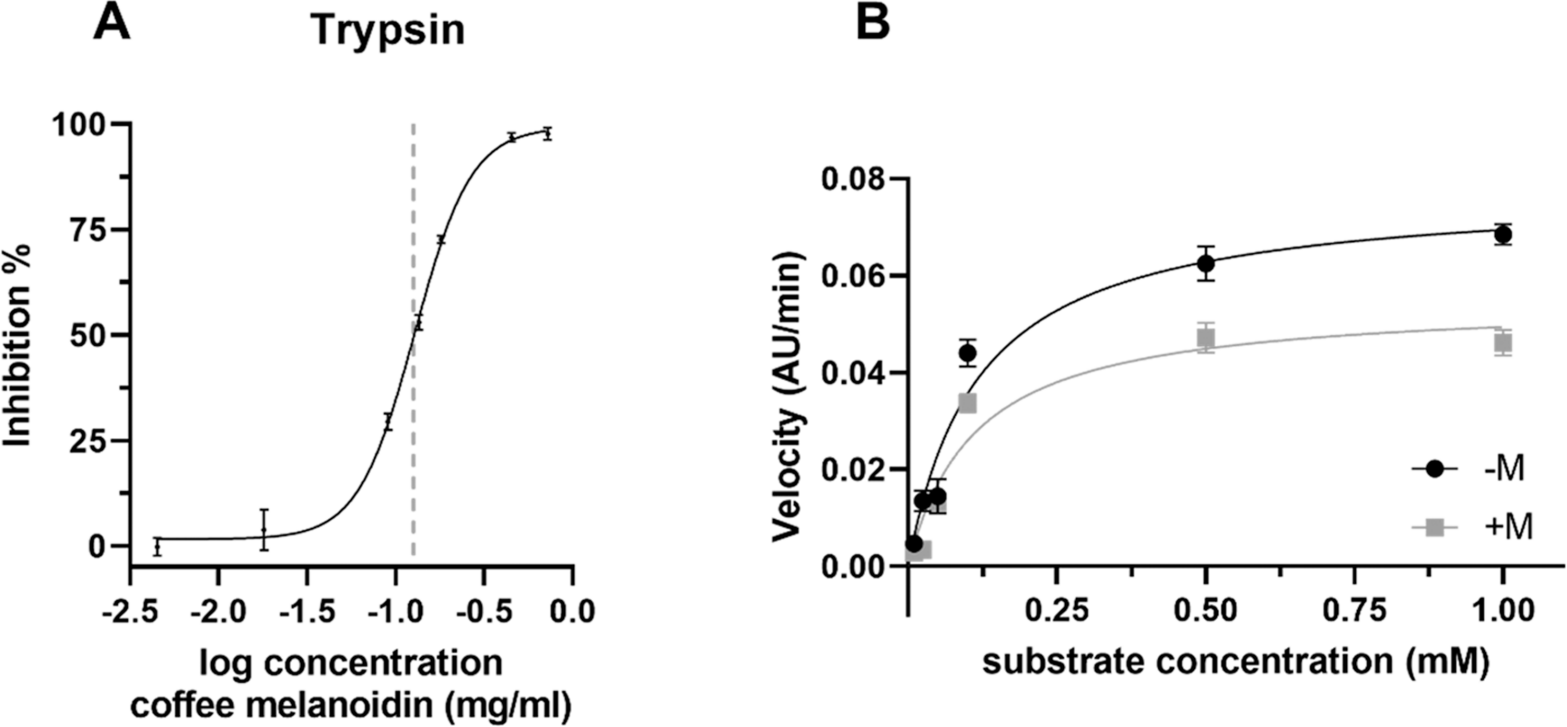
Inhibition of trypsin with water-soluble coffee melanoidins. (A) Dose–response curve of trypsin and water-soluble coffee melanoidins. The dotted line marks the concentration present in the simulated intestinal digestion experiment (0.125 mg/mL). (B) Michaelis–Menten kinetics in the absence (−M) or presence of 0.08 mg/mL water-soluble coffee melanoidins (+M). Data are mean ± S.D., *n* = 3.

The inhibition of trypsin in the presence of coffee melanoidins ranging in concentration from 0.005 to 0.7 mg/mL was analyzed, and the results are shown in Figure 2A. In contrast to pepsin, trypsin activity is markedly reduced in the presence of coffee melanoidins. Based on the dose–response curve, the IC_50_ value of water-soluble coffee melanoidins is 0.123 ± 0.007 mg/mL. The relative volume proportion of the human stomach to the small intestine is 1:2.^30^ This means that food components undergo dilution when they are transferred from the stomach to the intestine. Based on the assumed concentration of coffee melanoidins in the stomach (see above), this would lead to a concentration of 0.15–0.625 mg/mL in the small intestine. Thus, coffee melanoidins in the small intestine can markedly inhibit trypsin activity in realistic dietary amounts.

To elucidate how coffee melanoidins affect trypsin activity, Michaelis–Menten kinetics analysis was performed (Figure 2B). Therefore, enzyme activity was recorded as a function of increasing concentrations of substrate in the presence or absence of coffee melanoidins at a concentration of 0.08 mg/mL.

The maximum velocity of the enzymatic conversion (*V*_max_) decreases from 0.08 to 0.05 AU/min in the presence of coffee melanoidins and cannot be recovered by the addition of more substrate. This indicates that the substrate and inhibitor (coffee melanoidins) have different binding sites. This is also confirmed by the Michaelis–Menten constant (*K*_m_). The *K*_m_ value of the Michaelis–Menten kinetics without an inhibitor is 0.1175, whereas the *K*_m_ value in the presence of coffee melanoidins is 0.1112. The constant *K*_m_ value shows that the affinity of the enzyme for its substrate stays the same in the presence or absence of coffee melanoidins, and no competition of the substrate with the inhibitor occurs. A decrease in *V*_max_ and a constant *K*_m_ value indicate a noncompetitive inhibition mode of coffee melanoidins on trypsin activity. It is rather unlikely that high-molecular-weight compounds, such as coffee melanoidins, can interact with the active site of trypsin. Other trypsin inhibitors originating from food sources such as soybeans and peas^31^ usually have a smaller peptide structure and commonly have a competitive mechanism for protease inhibition by binding and blocking access to the protease active site.^32^ Moreover, vegetable peptides can exert an emulsification effect which lowers overall protein digestibility.^33^ Protein digestibility and protein quality have been reported to be negatively affected in animal models by the presence of high levels of dietary trypsin inhibitors, mainly affecting limiting amino acids such as methionine, cysteine, and tryptophan.^34^ Due to their proteinaceous nature, plant-derived trypsin inhibitors can be inactivated by mechanical and thermal treatment (e.g., roasting, dehulling, blanching, soaking, and cooking), which are processing steps regularly applied during the manufacturing of plant-based food.^35^ Coffee melanoidins are high in non-carbohydrate and non-protein compounds (∼90%) and, as a result, only small amounts (less than 6% each) of releasable amino acids and carbohydrates can be determined.^36^ In addition, polyphenols and condensation products participate in the structural complexity of coffee melanoidins because of the transglycosylation reaction between galactomannans and arabinogalactans,^37^ isomerization reactions and hydrolysis of hydroxycinnamic acid residues (i.e., chlorogenic acid isomers).^38^ Since coffee melanoidin chemical structures strongly depend on the starting plant material and roasting conditions, it is difficult to speculate how these factors influence trypsin inhibition and whether changes in processing steps could alter the effect on trypsin activity. During roasting of coffee beans, one of the hypothesized reaction pathways encompasses polyphenols that generate quinic acid cross-links and, upon oxidation, quinones that in turn funnel the number of dicarbonyl-based reactive sites leading to the formation of reductones, polymers, and condensation products.^39^

**Figure 3:**
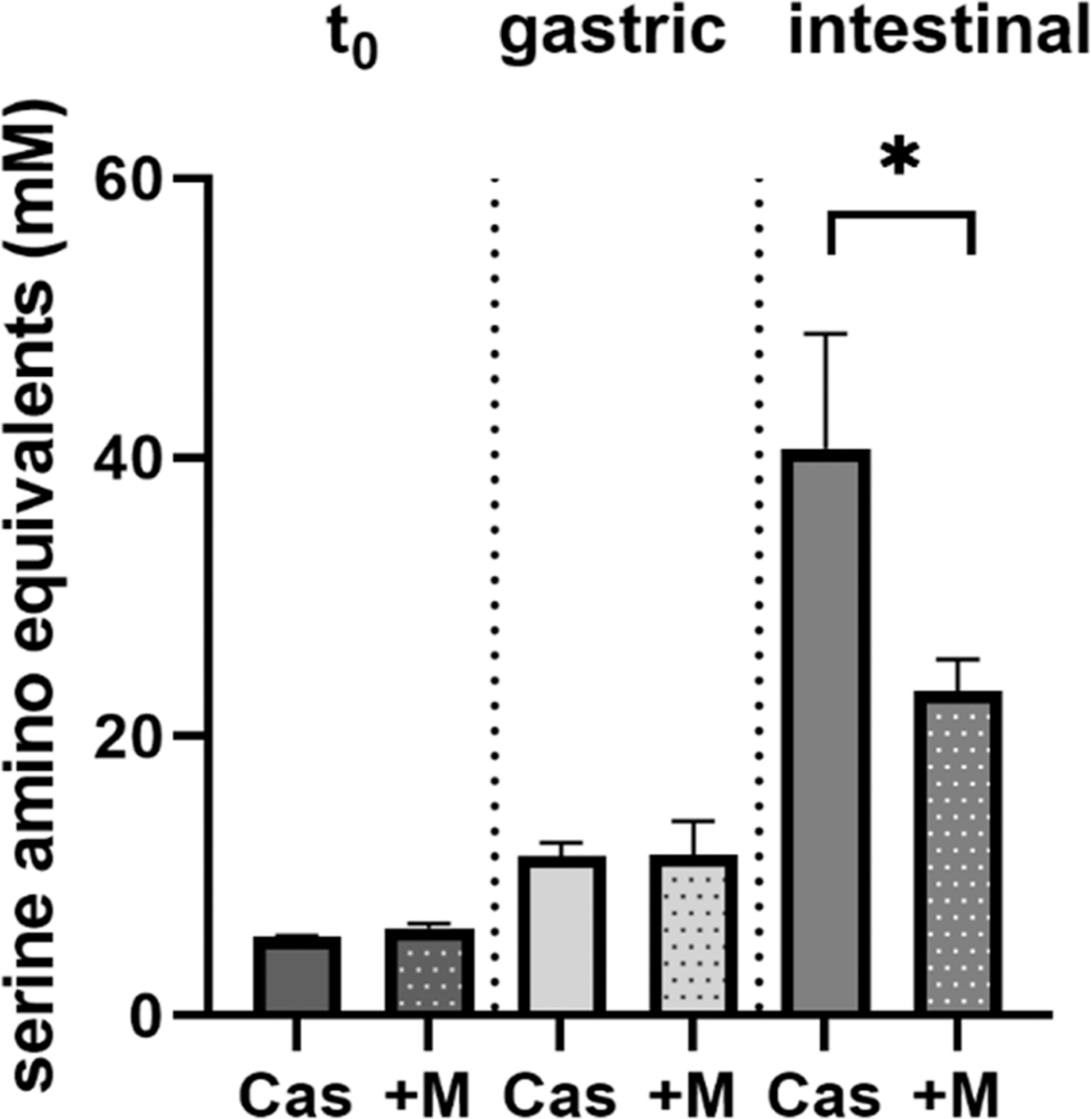
Release of amino compounds during simulated digestion in the presence or absence of water-soluble coffee melanoidins. The concentration of water-soluble coffee melanoidins at *t*_0_ is 0.5 mg/mL, in the gastric phase is 0.25 mg/mL, and in the intestinal phase is 0.125 mg/mL. Data are mean ± S.D., *n* = 3.

The enzyme activity assays used in this study to obtain dose–response curves do not mimic the conditions under which pepsin and trypsin are physiologically active. Therefore, the INFOGEST digestion model was used to simulate the gastrointestinal protein digestion of cow milk casein. Digestibility was evaluated based on the release of free amino compounds analyzed after derivatization with o-phthaldialdehyde (OPA) and subsequent absorption measurement. Blank values for the digestion without casein but simulated digestion fluids (enzyme blank) or without casein but melanoidins and simulated digestion fluids (melanoidin blank) were subtracted from the respective samples. Independent of the presence or absence of coffee melanoidins, the mean concentration of free amino compounds was approximately 6 mM before simulated digestion. This “background” amount of free amino compounds is not caused by a release of compounds during digestion but is rather due to derivatization of N-terminal amino groups of casein. The melanoidin blank showed serine amino equivalents of 0.1 mM, indicating that coffee melanoidins contain only a limited number of amino groups that form derivatives with OPA. After the addition of gastric fluids containing pepsin and subsequent incubation, the mean serine amino equivalents were 11.4 and 11.5 mM for gastric digestion of casein and casein in the presence of coffee melanoidins, respectively (Figure 3). The concentration of coffee melanoidins in the simulated gastric phase is 0.25 mg/mL and this concentration showed a pepsin inhibition of approximately 20% in the enzyme inhibition assay (see Figure 1, dotted line). However, the proteolytic activity of pepsin is not altered in the presence of coffee melanoidins during simulated digestion. After intestinal digestion, the mean release of free amino compounds from casein was 40.6 and 23.2 mM serine amino equivalents in the absence and presence of coffee melanoidins, respectively (Figure 3). In contrast to pepsin activity and gastric digestion, the inhibition of trypsin observed in the enzyme activity assay also translates into the inhibition of the release of amino compounds in the course of simulated intestinal digestion. The concentration of coffee melanoidins in the simulated intestinal phase is 0.125 mg/mL and this concentration showed a trypsin inhibition of approximately 50% (see Figure 2A, dotted line). Therefore, the inhibition of trypsin in the enzyme activity assay is comparable to the decrease in the release of amino compounds during intestinal digestion in the presence of coffee melanoidins.

**Figure 4:**
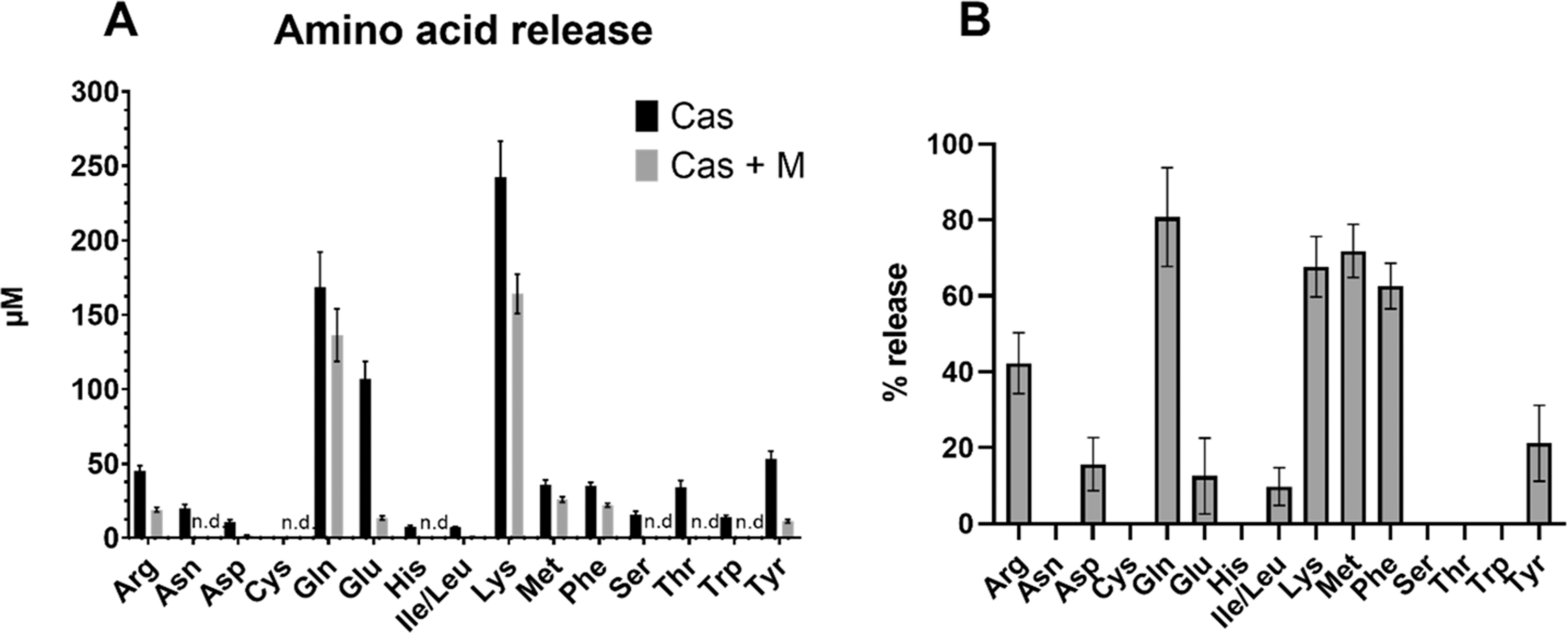
Release of amino acids after simulated intestinal digestion. (A) Absolute concentrations of amino acids after intestinal digestion of casein in the absence or presence of coffee melanoidins, measured according to analytical performances reported in Table 1 through pure analytical reference standards. (B) Release (%) of amino acids in the presence of coffee melanoidins. Data are mean ± S.D., *n* = 3, n.d. not detectable.

The decreased release of amino compounds during intestinal digestion in the presence of coffee melanoidins was further confirmed by analyzing the concentrations of amino acids through liquid chromatography with tandem mass spectrometry (LC-MS/MS). The sum of the released amino acids after intestinal digestion was 797 μM for casein and 394 μM for casein in the presence of coffee melanoidins. The spectrum of amino acids and how their release is affected by coffee melanoidins is shown in Figure 4A. Asparagine, cysteine, histidine, serine, threonine and tryptophan were only released in the absence of coffee melanoidins. Arginine, aspartic acid, glutamine, glutamic acid, leucine/isoleucine, lysine, methionine, phenylalanine, and tyrosine were also released during intestinal digestion in the presence of coffee melanoidins, but to a lesser extent (10–80% compared to individual casein digestion). With a release of ≤20% compared to the digestion of casein, the release of aspartic and glutamic acid, as well as isoleucine/leucine and tyrosine, was especially affected in the presence of coffee melanoidins (Figure 4B). Alanine, cysteine, glycine, proline, and valine were not released after casein digestion in the presence or absence of coffee melanoidins.

The implications of this newly discovered inhibitory effect of coffee melanoidins on gastrointestinal enzymes could be versatile. Caseins are highly sensitive to hydrolysis by digestive enzymes due to their chemical structure. Whether the digestibility of other food proteins with a less accessible structure, such as whey or soy protein, is affected in a similar manner by coffee melanoidins is another point worth of investigation. The presence of coffee melanoidins might lead to steric hindrance and to the formation of noncovalent interaction with casein that limits the accessibility of the digestive enzyme active site. Reduced proteolysis due to emulsion was demonstrated by Macierzanka et al.:^40^ an emulsion system of transglutaminase structured caseins significantly increased the resistance of the protein to pepsin leading to reduced proteolysis of high-molecular-weight cross-linked oligomers; conversely, this effect was not observed when cross-linked sodium caseinate was in solution. Whether reduced proteolysis in the presence of coffee melanoidins is due to multiphase microdispersed systems consisting of partially digested proteins and melanoidins needs targeted investigation. Moreover, a diet rich in melanoidins could lead to decreased protein digestibility resulting in higher amounts of undigested protein being transferred to the colon and being catabolized to aromatic, indole, and imidazole compounds, branched and short chain fatty acids, ammonia, amines, polyamines, and hydrogen sulfide.^41^ Whether these gut microbial metabolites are detrimental for health and linked to various disorders such as cancer, obesity, or diabetes is under debate.^42^ It has also been proposed that the intestinal exposure to food-derived protease inhibitors might be applicable for therapeutic use in the treatment of irritable bowel syndrome (IBS), a chronic GI disorder.^43^ Individuals suffering from IBS often report elevated protease activity in the intestinal mucosa or in stool samples, and elevated proteolytic damage on the extracellular matrix can often be seen in an inflamed gut. Whether food-derived protease inhibitors could alleviate this condition is unknown.

A limitation of the current study is the application of only one coffee sample used for the extraction of coffee melanoidins. It should be tested whether coffee melanoidins extracted from various coffee beans and different roasting conditions show similar results. This could also give information about a potential inhibition mechanism. Moreover, additional proteolytic enzymes, such as chymotrypsin and protein substrates different from caseins, should be studied to gain comprehensive knowledge regarding the inhibitory action of coffee melanoidins on gastrointestinal enzymes.

In this study, water-soluble coffee melanoidins were extracted from coffee brew and isolated by dialysis. It was shown that coffee melanoidins in concentrations which could be realistically achieved in the stomach with an average consumption of coffee decreased pepsin activity by 20–25%. The activity of the intestinal protease trypsin was affected more severely with an IC_50_ value of 0.12 mg/mL. Results of the in vitro INFOGEST protocol show that the release of free amino compounds from casein during gastric digestion is not affected by the presence of coffee melanoidins while the intestinal release was decreased to approximately 50%. The inhibition of proteolytic enzymes by coffee melanoidins might decrease the amount of essential amino acids available for intestinal resorption and, thus, decrease the nutritional value of proteins. Further studies are needed to confirm the results of this pilot study and to elucidate the physiological implications of trypsin inhibition by coffee melanoidins.
